# Aging and Microbiome in the Modulation of Vaccine Efficacy

**DOI:** 10.3390/biomedicines10071545

**Published:** 2022-06-29

**Authors:** Manoj Kumar, Meenu Mariya James, Manoj Kumawat, Bilkees Nabi, Poonam Sharma, Namrata Pal, Swasti Shubham, Rajnarayan R. Tiwari, Devojit Kumar Sarma, Ravinder Nagpal

**Affiliations:** 1 National Institute for Research in Environmental Health, Bhopal 462030, India; manoj15ndri@gmail.com (M.K.); meenumariya07@gmail.com (M.M.J.); manojkbiochem@gmail.com (M.K.); poonam.mannan91@gmail.com (P.S.); namratapal017@gmail.com (N.P.); swasti.shubham@gmail.com (S.S.); rajtiwari28101@yahoo.co.in (R.R.T.); 2 Department of Biochemistry and Biochemical Engineering, Sam Higginbottom University of Agriculture, Technology and Sciences, Allahabad 211007, India; bilkeesmir90@gmail.com; 3 Department of Nutrition and Integrative Physiology, Florida State University, Tallahassee, FL 32302, USA

**Keywords:** aging, gut microbiome, host-immune system, vaccine response, COVID-19, pandemic

## Abstract

From infancy through to old age, the microbiome plays an important role in modulating the host-immune system. As we age, our immune system and our gut microbiota change significantly in composition and function, which is linked to an increased vulnerability to infectious diseases and a decrease in vaccine responses. Our microbiome remains largely stable throughout adulthood; however, aging causes a major shift in the composition and function of the gut microbiome, as well as a decrease in diversity. Considering the critical role of the gut microbiome in the host-immune system, it is important to address, prevent, and ameliorate age-related dysbiosis, which could be an effective strategy for preventing/restoring functional deficits in immune responses as we grow older. Several factors, such as the host’s genetics and nutritional state, along with the gut microbiome, can influence vaccine efficacy or reaction. Emerging evidence suggests that the microbiome could be a significant determinant of vaccine immunity. Physiological mechanisms such as senescence, or the steady loss of cellular functions, which affect the aging process and vaccination responses, have yet to be comprehended. Recent studies on several COVID-19 vaccines worldwide have provided a considerable amount of data to support the hypothesis that aging plays a crucial role in modulating COVID-19 vaccination efficacy across different populations.

## 1. Introduction

The current COVID-19 pandemic has pressed the scientific society internationally to develop vaccines against SARS-CoV-2. Several vaccine candidates have hitherto been developed to combat the COVID-19 pandemic. To date, over 20 vaccines have been approved for emergency use in different countries, while several are still in the clinical trial stage. These vaccine candidates are based upon inactivated protein sub-units, non-replicating viral vectors, and DNA and RNA elements. Vaccines are the most effective means of preventing infectious diseases; however, vaccine-induced immune responses have been found to vary greatly amongst people and communities in different parts of the world. This disparity underscores the utmost importance of comprehensive understanding of the basis of this variable immune response. Although there are many factors that may influence intra- and inter-population heterogeneity in vaccine response [1], increasing evidence suggests that the host’s genetics, age, gut microbiome, nutritional status, and ethnicity play a role in regulating immunological response to vaccination [2]. Several studies on infant cohorts have shown that the immune responses are more prominent in high-income nations than in low/middle-income countries (LMICs). This is particularly applicable in developing countries, mainly due to demographic shift and the changes in lifestyle from a nomadic to an urban one, which may have a role in gut dysbiosis [3]. For instance, the Bacillus Calmette-Gue’rin (BCG) tuberculosis vaccine response rate is higher in Europe than in Africa [1,4]. Vaccines against other diseases, such as rotavirus, poliomyelitis, yellow fever, and malaria, have been found to show a similar tendency to provide less response/protection in Africa and Asia, compared to Europe and the United States [5,6,7,8]. Frequent observations have also recorded less immune response against oral vaccines for polio and rotavirus in LMICs, compared to developed countries [9,10,11,12,13,14].

The current COVID-19 pandemic is an unprecedented event, wherein such a large adult population worldwide, with diverse ages, ethnicities, and dietary habits, is undergoing vaccination. Given that the adult population is also susceptible to comorbidities such as diabetes, high blood pressure, etc., there is a need to check vaccine responses, as they provide a unique opportunity to explore the impact of gut microbiota, nutritional status, and ethnicity on immunological response to vaccination. We hypothesize that an individual’s gut microbiota and nutritional status may facilitate COVID-19-specific immune cell expansions, thereby modulating vaccine immunogenicity and efficacy; this effect could be assessed by an integrated analytical approach, including microbiome characterization, nutritional status, and vaccine immunological response, with the help of a system biology approach. Several studies have explicitly analyzed the role of gut microbiota (as a whole or in terms of important microbial taxa) and host nutritional status in vaccine immunological responses. Gut dysbiosis (imbalanced gut microbiota), which is tightly linked to the host’s nutritional state along with several other host-specific factors, can inhibit immune responses, thereby negatively impacting vaccine efficacy. Hence, determining the role of the gut microbiome and the host nutritional status in COVID-19 vaccine responses in different ethnic populations, particularly from LMICs and developed countries, may help in identifying key nutrients and microbial colonization patterns in relationship to immune training in adults, paving the way for targeted immune augmentation strategies, as well as identifying novel targets for interventions aimed towards boosting vaccination response. It may also be particularly important for LMICs countries to check and monitor the response of COVID-19 vaccine candidates before finalizing plans for mass immunization, and to select a specific vaccine candidate for which the trials are done successfully in a specific country.

## 2. Influence of Gut Microbiome on Vaccine Response and Efficacy in LMICs

The gut microbiome composition and functions play a critical role in host vaccine response efficacy and disparity among different individuals and populations. Various mechanisms—including the natural adjuvant hypothesis; microbial metabolites modulating B cell responses; and vaccination antigen cross-reactive microbiota-encoded epitopes—have been implicated in these effects [15]. Immunomodulatory molecules, such as flagellin and peptidoglycan, produced by the microbiota are natural adjuvants that can be recognized by antigen-presenting cells (APCs), such as pattern recognition receptors (PRRs), toll-like receptors (TLRs), and nucleotide-binding oligomerization domain-containing protein-2 (NOD2) [15].

Lipopolysaccharides (LPS), a major constituent of the outer layer of Gram-negative bacteria, are also a powerful innate immune stimulator. They consist of a poly- or oligo-saccharide region attached to a carbohydrate lipid known as lipid A, which is the main immune stimulatory center of LPS [16]. The structure of LPS and lipid A differs from species to species, and this variation has a substantial effect on the immune stimulatory capacity of that particular species [17]. Studies on the immunogenicity of diverse gut bacteria species have revealed that, in comparison to *E. coli*-derived LPS, *Bacteroidetes*-derived LPS have an impaired or even immune-inhibitory capacity to induce inflammatory cytokines [18]. An association has also been observed between the oral Rotavirus vaccine response and the gut microbiome in infant cohorts from Ghana and the Netherlands, wherein the rotavirus vaccine response was found to be correlated with a reduced abundance of *Bacteroidetes* and an increased abundance of *Streptococcus bovis* belonging to the *S. bovis–Streptococcus equinus* complex, along with the *E. coli*, *Serratia*, and *Klebsiella* groups [19]. The LPS of *S. bovis–S. equinus* complex bacteria can trigger inflammatory responses by acting as natural vaccine adjuvants. Moreover, in the vaccine response group, the serum IgA was positively associated with *Streptococcus bovis* abundance, and negatively associated with the abundance of *Bacteroidetes* [19,20]. In a parallel study of infant cohorts from Pakistan compared to the Dutch cohorts, a notable positive association was observed between Rotavirus vaccine response and a lower abundance of *Proteobacteria*, particularly *Serratia* and *Escherichia coli* in the non-responders. The post-vaccination serum IgA level was also observed to be associated with an abundance of *Proteobacteria*. Because *E. coli*-derived LPS has a higher immune-stimulatory potential than LPS derived from other microbial phyla, such as *Bacteroidetes*, the presence of these species in the vaccine response group may boost the innate and adaptive immune responses [21]. These findings support the natural adjuvant hypothesis as a potential mechanism by which gut microbiota may modulate the vaccine response [15].

A prospective observational study of oral polio virus (OPV), BCG, tetanus toxoid (TT), and hepatitis B virus (HBV) vaccines in Bangladeshi infants, specified that the relative abundance of phylum Actinobacteria (family *Corynebacteriaceae* and family *Bifidobacteriaceae*) was positively associated with high OPV CD4 T-cell response, OPV-specific IgG response, and BCG-CD4 T-cell response to PPD antigen. Furthermore, the abundance of family *Bifidobacteriaceae* was positively associated with high OPV-CD8 response and TT-CD4 response, while the abundance of the order *Actinomycetales* was positively associated with TT-IgG response. Only the genus *Rothia* was observed to be positively corelated with high HBV-IgG response [22]. Similarly, a positive association of *Bifidobacterium* abundance in early infancy with memory responses to early vaccination was observed in a parallel study conducted on infant cohorts in Bangladesh. Infants were vaccinated at various time periods, viz: BCG (at birth); OPV (at the time of birth, or at 6, 10, and 14 weeks); TT (at 6, 10, and 14 weeks); and HBV (at 6, 10, and 14 weeks). The abundance of *Bifidobacterium* in early childhood was linked to CD4^+^ memory T-cell responses to BCG. A comparable positive link was seen between the abundance of *Bifidobacterium*, namely *B. longum*, and IgG responses towards the TT vaccine at 15 weeks and 2 years. Plasma IgA and plasma IgG concentrations at 2 years also had a positive association with *Bifidobacterium* abundance [23]. A similar study conducted in Bangladesh evaluated the safety and enhancement of vaccine response by the administration of a probiotic strain, *Bifidobacterium breve* BBG-01, along with the oral inactivated cholera vaccine. It was observed that the serum LPS-specific IgA and fecal cholera toxin B (CTB)-specific IgA, present in the BBG-01 group of infants, was greater in proportion in 37–48-months-old and 49–60-months-old infants, respectively. The negative correlation noticed between *Bifidobacterium* and *Enterobacteriaceae* in the BBG-01 group suggests a conceivable contribution of the probiotic strain BBG-01 to alteration of the bacterial ecology of the intestine and enhanced vaccine response among the probiotic group [24]. However, the interaction between the probiotic *Bifidobacterium* strains and the indigenous bifidobacterial population remains to be explicated; this should be an important topic for further studies to compare the administration of specific *Bifidobacterium* strains supplemented with the vaccine with the presence of other bifidobacterial species and strains in the gut. Another study conducted in Nicaragua observed that the sero-converters of the oral pentavalent Rotavirus vaccine had a higher relative abundance of *Bacteroides caccae* and *Fusobacteria*, with a ≥4-fold rise in RV-specific IgA titers post-vaccination [25]. In a similar study conducted in Zimbabwe, a significant positive correlation between anti-rotavirus IgA titer and *Bacteroides thetaiotaomicron* abundance was observed in infants [26]. The clinical evidence from the observational and interventional studies conducted in LMICs and HICs is summarized in Table 1, depicting how intestinal microbiota may play a critical role in host vaccine response and efficacy. This may be particularly relevant to the relatively reduced or compromised nutritional status of the LMICs populations, which could make them more predisposed to gut microbiome dysbiosis and the consequent dysregulated vaccination response.

## 3. Innate, Adaptive, and GALTs-Associated Immunity in Aging

**Figure 1:**
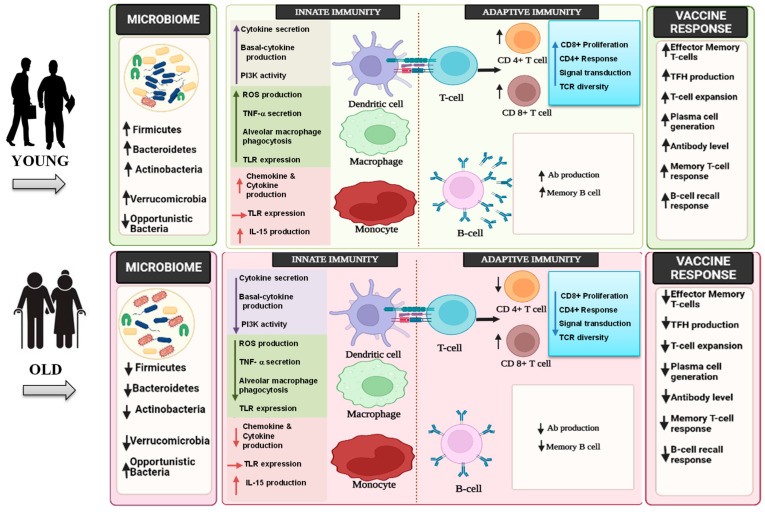
Aging and vaccine response: gut microbiota vis-à-vis innate and adaptive responses. ROS: reactive oxygen species; TLR: toll-like receptors; IL: interleukin; TNF: tumor-necrosis factor; Ab: antibodies; TCR: T-cell receptor; TFH: T-follicular helper cells; ↑: higher/increased; ↓: lower/decreased.

In general, the innate immune system exhibits dysregulated inflammatory responses with aging, which can result in a pro-inflammatory state in humans. Such inflammatory processes may lead to failure of innate immune activation against infections or vaccinations. Although the impacts of aging on the innate immune system need more decipherment, there is adequate evidence documenting the decline of immunological functions with aging (Figure 1). The immune memory is a critical component of the immune system, as it allows the host to have a quick and robust reaction to the pathogen when it is encountered for the second time, thereby laying the groundwork for vaccination. This immune-based mode of action of vaccines is an important preventative tool against illnesses; however, the responses to vaccination, in terms of antibody titer, effectiveness, and affinity of antibody generated, have been reported to decline with age.

Innate immunity serves as the first line of defense against infections infiltrating our body. The cells engaged in this process are neutrophils, monocytes, macrophages, and dendritic cells, which all interact with adaptive immunity. These cells are formed and mature at different times during fetal life, and the function of all innate immunity components is poor in neonatal years in comparison to later years of life. The phagocytic property of macrophages from aged persons has been found to be diminished with lower levels of macrophage-secreted chemokines—viz., MIP-1α, MIP-1β, MIP-2, and eotaxin—and also to reduce the level of phosphatidylinositol-2-kinase protein B (PI3K-AKT) and cGMP-AMP synthase stimulator of interferon genes (cGAS—STING) signaling [31,32,33].

Studies have also shown that the ssRNA virus, West Nile Virus (WNV), exhibits enhanced pathogenicity in older individuals, which is similar to SARS-CoV-2. According to these studies, infection with WNV causes TLR3 to be down-regulated in monocyte-derived macrophages from older donors. This mechanism involves poor signaling of DC-SIGN and STAT-1, leading to higher and longer-lasting cytokine levels, which could explain why WNV is more severe in older people [34,35]. Moreover, the majority of studies have observed no difference in the overall number of monocytes with aging during baseline state, but have discovered a rise in the number of CD14dimCD16+ monocytes with a reduced response to PRR ligation [36,37,38]. Similar studies investigating the transcriptional and functional variations of monocyte subsets among young and elderly persons analyzed that, without stimulation at baseline, the surface expression of TLR3, TLR4, and TLR 7 was analogous among young and old age groups. On the other hand, in all age groups, the transcription and synthesis of critical pro-inflammatory cytokines (IFNγ, IFNα, IL-1β) and crucial recruiting chemokines (CCL20 and CCL8) were reduced after activation with agonist to these TLRs [39]. Dendritic cells are essential for the initiation and modulation of immune responses, but their significance in bridging innate and adaptive immunity in humans is not well understood. When compared to young donors, primary mDCs (cDC1) and pDCs had reduced levels of IL-6, IL-12, and TNF-, as well as relatively low surface expression of TLRs, but higher basal levels in response to TLR 3, TLR 7, TLR 8, and TLR 9 ligand activation [40]. Other studies have found that pDCs are less frequent in older people and produce less type-I IFN in response to IAV infection and WNV, although other DC populations are conserved [41]. Studies have also reported reduction in expression of CD80/CD86 and in production of type-I IFN in monocyte-derived DCs (MDDCs) after contracting WNV infection. This change is linked to lower STAT1 and IRF 7 expression, which is also observed in primary pDCs [42,43]. It has also been found that infection with SARS-CoV-2 results in decrease in DCs development and consequent T-cell-mediated reactions [44,45].

Various cell lineages that regulate innate immunity exhibit a wide range of aging characteristics that represent their developmental, tissue, and activation contexts. Overall, research in aged mice (20-months-old) and humans (≥65-years-old) has shown that activation of the innate immune system produces dysregulated inflammation in the elderly, which is characterized by elevated basal inflammation as well as a reduction in effective innate and adaptive immune responses to pathogens or vaccination antigens [46]. However, more research is needed to analyze if a poor vaccine response may be improved with the addition of an agonist. Age-related declines in TLR, RLR, and inflammasome pathways expression and activation may lead to deficiencies in viral infection responses, which may result in age-related susceptibility to viruses such as IAV and now SARS-CoV-2.

Pertaining to the adaptive immune system remodeling with aging, T-cells show progressive reduction with age. The shift in T-cells with aging can be connected to the involution of the thymus, which results in differences of naive and memory T-cells proportions, resulting in a skewing toward memory T-cells in older persons [47,48,49]. With advanced age, CD8+ T-cells exhibit a large decline in the number of naive T-cells in the blood; and both CD4+ and CD8+ T-cells demonstrate a significant reduction in naive T-cell frequency in secondary lymphoid regions [46]. With the reduction in the overall number of naive T-cells with age, a decrease of TCR clonal diversity, particularly that of CD8+ T-cells, occurs [50,51]. Furthermore, the Th1 and Th2 responses are also altered with aging, while other subsets of helper T-cells, such as CD4+ T-follicular helper (Tfh) and T-regulatory cells, are similarly influenced by aging. It has also been shown that the frequency of circulatory Tfh corresponds to low levels of IgG in older adults when co-cultured with the B-cells of young adults [52]. Besides the apparent abnormalities in circulatory Tfh cells, chronic activation in older persons can also lead to cell depletion, thereby leading to a poor response to vaccination. Tregs, on the other hand, become more frequent in older age, although they seem to retain their functionality as people grow older [53]. This rise in Treg frequency and activity in older adults could further dampen the immunological response mediated by CD8+ and CD4+ T-cells, which is already diminishing in older adults [54]. This impairment could contribute to the lower vaccination efficacy which is observed in older persons, as it could hinder a significant immunological response [55]. Notably, emerging evidence also suggests a role for T-cell based immunotherapy in diseases beyond cancer, such as autoimmune disorders and viral infections, including SARS-CoV-2 [56].

Furthermore, B-cells also appear to show progressive alterations during the aging process. The overall number of B-cells reduces progressively with age, and their diversity also declines due to clonal expansion with age, which is similar to how T-cell diversity decreases with age [57,58]. It has been reported that the percentage of naive B-cells significantly increases in CD19+ cells, which probably leads to a sharp fall in particular memory B-cell subsets. While the fraction of IgM+ memory B-cells appears to remain constant with age, the number and percentage of IgG+ and IgA+ B-cells and plasma blasts (PB) decrease dramatically [59]. Some studies have postulated that the reduction in AID (an enzyme that is essential for both isotype switching and somatic hypermutation) in B-cells might be a sign of poor vaccine response in older persons, as the reduced ability to class switch limits the defensive humoral response to immunization [60,61].

**Figure 2:**
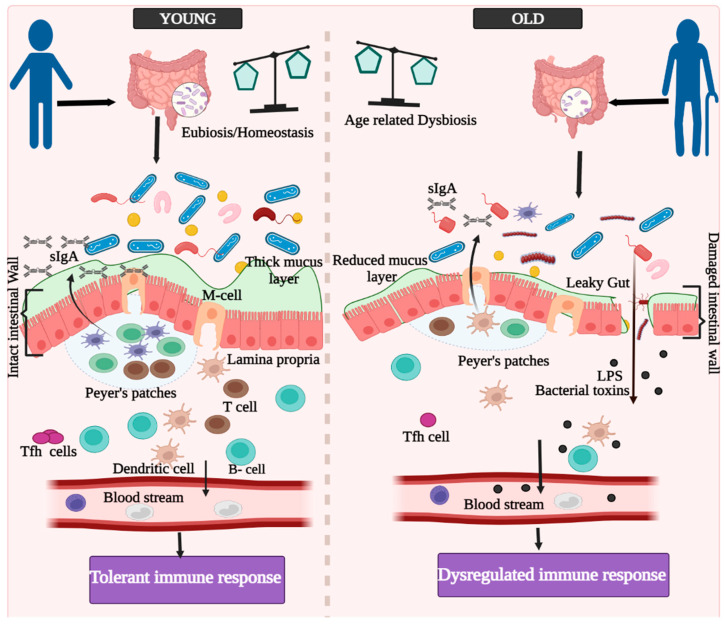
Aging-associated gut dysbiosis and GALT-associated immune responses in the elderly with ‘leaky’ gut. Tfh: T-follicular helper cells; LPS: lipopolysaccharide; sIgA: secretory immunoglobulin A.

GALT (gut-associated lymphoid tissue) is made up of two distinct lymphoid structures [61]. Peyer’s patches (PPs) and lymphoid follicles are seen throughout the small and large intestine tract, including the immunological defense system, and have been identified as sites for the development of intestinal secretory-immunoglobulin A (IgA) responses (Figure 2). The gastrointestinal immune system’s PPs are one of the most well-organized lymphoid tissues [62,63,64,65]. SIgA (secretory IgA) binds to the biologically active regions of gut pathogen-produced toxins, and neutralizes them. The gut contains around 20% of all lymphocytes [66], which are exposed to a variety of exogenous immunogens. Gut immune cells stay updated on the frontier with a potentially harmful infection source. Gut bacteria have an impact on the development of Th17 [67], Treg cells [68], and also memory T-cells [69,70]. These immunological properties of the gastrointestinal tract have propelled researchers to develop efficient oral vaccinations. Most notably, oral vaccination can result in both mucosal and systemic immunity, resulting in a protective immune response with two levels [71].

## 4. Aging and Immunosenescence in the Context of Vaccine Responses

Recent molecular and cellular studies have revealed the crucial role of aging in the instabilities instigated in the host immune system, such as inflammation and metabolic dysfunctions [72]. Physiological mechanisms such as senescence, i.e., the gradual diminishing of cellular functionalities, affecting the aging process, and the relatively declined vaccine responses, are yet to be explored. The recent trials of various COVID-19 vaccines worldwide have yielded a substantial quantity of data hypothesizing that the aging process has a crucial role in the modulation of COVID-19 vaccine efficacy across different populations.

A recent study on the immunogenicity and efficacy of Pfizer-BioNTech’s BNT162b2 COVID-19 vaccine in adolescents found that the serum-neutralizing geometric mean titer (GMT) in the 16–25-year-old group, after one month of the second dose, was relatively low compared to that in the 12–15-year-old group [73]. A phase 1/2 trial for detecting the immunogenicity and safety of BBIBP-CorV COVID-19 vaccine among the Chinese population aged 18 years and above, by categorizing into 2 μg, 4 μg, and 8 μg vaccine dose cohorts, observed that in all the cohorts, the neutralizing antibody GMTs were significantly less in the ≥60 years age group [74]. A phase I clinical trial of a recombinant COVID-19 vaccine (V-01), including fusion protein, categorized the Chinese experimental population into healthy younger individuals (18–59 years) and elder persons (60 years) (IFN-PADRE-RBD-Fc dimer), and observed that both the neutralizing antibody GMTs and the RBD-binding capacity GMT were low in the elder adult cohort compared to the younger adult cohort [75]. In a Russian study, the efficacy of Gam-COVID-Vac (rAd26 and rAd5 vector-based prime boost) among the healthy population was observed to be 91.9% in 18–30 years, 90% in 31–40 years, 91.3% in 41–50 years, 92.7% in 50–60 years, and 91.8% in >60 years [76]. A randomized clinical trial on the safety and immunogenicity of a denatured SARS-CoV-2 vaccine, CoronaVac, in the Chinese population aged ≥ 60 years, revealed a negative association between the neutralizing antibody GMTs and the increase in age group. In both the phases of the trial, ≥70 years showed relatively less neutralizing antibody GMTs, compared to the 60–64 years and the 65–69 years age groups [76]. A parallel study on the immunogenicity of Moderna’s SARS-CoV-2 mRNA-1273 vaccine in the older USA population, aged above 56 years, obtained a similar result wherein the neutralizing antibody GMTs after 57 days of vaccine administration were significantly higher in the 56–70 years age group in comparison with the ≥71 years age group [77]. A noteworthy difference in the vaccine response was seen between 18–55 years and ≥55 years age group participants, where less neutralizing antibody GMTs were detected in the ≥55 years age group at day 29, day 43, and day 57 after Moderna’s mRNA-1273 vaccine administration [78]. Similarly, the efficacy of Moderna’s mRNA-1273 among the study population of the USA was observed to be 95.6% in 18–65 years and 86.4% in ≥65 years with a 100 μg dose of the vaccine [79]. A phase 2/3 trial among the young and old adults of the UK revealed that, in both the low dose group and the standard dose group, the ≥70 years age group resulted in low anti-spike IgG responses for ChAdOx1 nCoV-19 vaccine compared to the 18–55 years and 56–69 years age groups (3565 AU/mL, 6439 AU/mL, 4553 AU/mL in low dose and 4156 AU/mL, 9807 AU/mL, 5496 AU/mL in standard dose group, respectively) [80]. A contradictory observation was made by a clinical trial on the immunogenicity of Pfizer-BioNTech’s BNT162b2 in the 18–55 years and 65–85 years age groups, wherein comparable dose-dependent SARS-CoV-2–neutralizing GMTs were shown by both the age groups [81].

The available scientific evidence obtained from the clinical trials on the COVID-19 vaccines depicts that there is a significant reduction in vaccine efficacy and response with advanced aging. This can be correlated with immunosenescence, inflammaging, and gradual fluctuation in diversity, composition, and function of the gut microbiome. As the gut microbiome has a mutualistic relationship with the host immune system, any deviations in its composition can be correlated with the deterioration of immune functioning, thereby leading to a reduced vaccination response [82].

## 5. Conclusions and Future Perspectives

Human gut microbial colonization begins at birth (or perhaps even before that), and keeps on fluctuating with the aging process. It is, however, found to be most deviant and unstable during infancy and old age. Behavioral factors such as smoking, alcohol consumption, and physical activity; environmental factors including geographical location and pollution; intrinsic host factors like genetics and comorbidities; and nutritional factors and dietary habits, are the potential factors affecting gut microbial functioning resulting in impaired vaccine immunogenicity. A drastic difference can be observed between LMICs and HICs regarding the factors affecting gut microbiome. The observational and interventional studies conducted to date describe a significant correlation between the differences in gut microbial diversity and abundance and the differences in vaccine immunogenicity among infants and children in LMICs and HICs. The impaired vaccine responses in LMICs for BCG, DTP–HepB–Hib, tetanus, and hepatitis B virus are affected by numerous factors that subsequently modify the gut microbiome makeup and function. Age-related gut dysbiosis occurs through the loss of beneficial microbes, including *Clostridia* and *Bifidobacterium*, and an increase in the number of *Proteobacteria*, wherein several pathobionts such as *Enterobacteriaceae* may predispose the elderly to various infectious diseases, especially the current COVID-19 pandemic. Given that gut microbiome alterations leading to gut dysbiosis may affect vaccine immunogenicity in infants and children, further investigations are essential, in order to comprehend the effect of gut dysbiosis in aged populations. As most vital immunizations have been administered by the age of 15, the exact influence of the gut microbiome on vaccine responses among adults and the elderly, is not accurately understood. A better understanding of aging-related immunosenescence, and its implications related to different vaccine responses—especially responses for COVID-19 vaccine candidates—is essential, in order to providing a wide-reaching impact on mass vaccinations. Since most developing countries are facing a demographic shift towards the late expanding stage, the number of aged populations is significantly increasing. Therefore, further studies are imperative, focusing on the elderly population, with respect to aging-related immunosenescence, inflammaging, gut dysbiosis, and modulation in vaccine response and efficacy, in order to ensure the success of mass immunization across all age groups.

## Figures and Tables

**Table 1:** Evidence of the influence of the Gut Microbiome on vaccine response in children.

Country	Study Design	Vaccine	Participant Specifications	Study Method	Vaccine Response and Gut Microbial Signatures	Reference
Bangladesh	Prospective observational study	OPV BCG TT HPV	48 infants 6, 11 and 15 weeks of age	16S ribosomal RNA sequencing Terminal restriction fragment length polymorphism (T-RFLP) assay Lymphocyte supernatant assay	**Phylum *Actinobacteria* (family *Corynebacterium*) abundance positively associated with:** High OPV CD4 T-cell response High OPV specific IgG response High BCG-CD4 T-cell response to PPD antigen **Phylum *Actinobacteria* (family *Bifidobacteriaceae*) abundance positively associated with** High OPV-CD4 T-cell response High OPV-CD8 response High OPV-IgG response High BCG-CD4 T-cell response to PPD antigen High TT-CD4 response **Phylum *Actinobacteria* (order *Actinomycetales*) abundance positively associated with:** TT-IgG response **Phylum *Actinobacteria* (genus *Rothia*) abundance positively associated with:** High HBV- IgG response	[22]
Bangladesh	Prospective Observational Study	BCG OPV TT HPV	Infants of 6, 11, and 15 weeks: (*n* = 291) Infants at 2 years: (*n* = 249)	ELISA Sequencing of 16s V4 region	***Bifidobacterium* abundance positively associated with:** CD4 T-cell responses to BCG, TT, and hepatitis B virus at 15 weeks CD4 responses to BCG and TT at 2 years plasma TT-specific IgG at 2 years stool polio-specific IgA at 2 years.	[23]
Ghana Netherlands	Nested, case-control study	Rotarix^TM^ (Rotavirus vaccine)	6–14 weeks old infants Responders (*n* = 30) Non-responders (*n* = 30) Healthy Dutch infants: (*n* = 154)	ELISA HIT (Human Intestinal Tract) Chip microarray analysis	**Abundance of *Streptococcus bovis* positively associated with:** RVV specific serum IgA **Abundance of *Bacteroidetes* negatively associated with:** RVV specific serum IgA	[19]
Pakistan Netherlands	Nested Matched Case-Control Study	Rotarix^TM^ (Rotavirus vaccine)	6–14 weeks old infants Responders: (*n*= 10) Non-responders (*n* = 10)	ELISA HITChip microarray analysis	**Abundance of *Clostridium* cluster XI and *Proteobacteria* (*Serratia* and *Escherichia coli*) positively associated with:** RVV specific serum IgA	[21]
Zimbabwe	Randomized, controlled trial	Oral rotavirus vaccine (Rotarix^TM^)	6–10 weeks of age Non-seroconverters (*n* = 81) Sero-converters (*n* = 34) Seronegative (*n* = 113) Seropositive (*n* = 45)	ELISA Whole metagenome shotgun sequencing	**Abundance of *Bacteroidetes thetaiotaomicron* positively associated with:** anti-rotavirus IgA titre	[26]
Bangladesh	Randomized, controlled trial	Oral cholera vaccine	2–5 years BBG-01 (*Bifidobacterium breve* and OCV; *n* = 64) Placebo group (placebo and OCV; *n* = 62)	ELISA RT-qPCR	**BBG-01 group:** Serum LPS-specific IgA present in a significantly higher proportion in the 37–48 months old infants. Fecal cholera toxin B (CTB)-specific IgA present in a significantly higher proportion in 49–60 months age subgroup.	[24]
Nicaragua	Randomized, controlled trial	ORV (RotaTe, RV5, Merck, Kenilworth, NJ)	2 months Sero-converters (*n* = 25) Non-sero-converters (*n* = 20)	ELISA 16S rRNA amplicon sequencing	**Sero-converters (≥4-fold increase in RV-specific IgA titers post-vaccination):** Higher relative abundance of *B. caccae*, *Fusobacteria*. Lower relative abundance of family *Enterobacteriaceae.*	[25]
India	Nested case–control study	ORV (Rotarix^TM^)	6–10 weeks of age **Entero-pathogen subset:** Responders (*n* = 162) Non-responders (*n* = 163) **Microbiota subset:** Responders (*n* = 85) Non-responders (*n* = 85)	TaqMan array card testing for entero-pathogens Sequencing of V4 region the 16S rRNA gene	No significant association	[27]
Blantyre (Malawi) Vellore (India) Liverpool (UK)	Prospective cohort study	Oral rotavirus vaccine (Rotarix^TM^)	Indian cohort (*n* = 307); 6–10 weeks Malawi cohort (*n* = 119); 6–10 weeks UK cohort (*n* = 60); 12 weeks	ELISA 16S rRNA amplicon sequencing	Negative correlation of *Sutterella* with dose 1 ORV shedding in Malawian infants. *Dolosigranulum*, *Corynebacterium* and *Lactobacillus* were negatively correlated with ORV shedding and final RV-IgA concentration in India.	[28]
India	Randomized, controlled trial	Oral poliovirus vaccine (OPV)	6–11month old infants **Seroconversion group** Seropositive (*n* = 62) Seronegative (*n* = 52) **Shedding group** Shedders (*n* = 42) Non-shedders (*n* = 33)	TaqMan array card testing Sequencing of V4 region the 16S rRNA gene	The abundance of class *Clostridia* significantly correlated with the OPV non-shedding group.	[29]
China	Randomized, controlled trial	Combination of inactivated polio vaccine (IPV) with OPV	2 months old infants **Polio-specific IgA-positive (IgA.P) infants:** day 28 (*n* = 66) day 14 (*n* = 65) day 0 (*n* = 66) **Polio-specific IgA-negative (IgA.N) infants:** day 28 (*n* = 39) day 14 (*n* = 38) day 0 (*n* = 39)	16S ribosomal RNA sequencing ELISA	**Baseline abundance of gut microbe at the time of receiving 1st dose of OPV:** IgA.P infants: phylum *Actinobacteria* was significantly enriched. IgA.N infants: phylum *Firmicutes* was significantly enriched. **After OPV administration:** IgA.N infants: the class *Clostridia* and *Firmicutes* was relatively abundant IgA.P infants: the class *unidentified, Actinobacteria* and *Bifidobacterium* showed higher abundance.	[30]

## Data Availability

Not applicable.
